# Supplementary material for: Human genomic regions with exceptionally high levels of population differentiation identified from 911 whole-genome sequences
Source: Genome Biol. 2014 Jun 30;15(6):R88. doi: 10.1186/gb-2014-15-6-r88 (PMC4197830; doi:10.1186/gb-2014-15-6-r88)

Additional figures for:

Human genomic regions with exceptionally high or low levels of population differentiation identified from 911 whole-genome sequences

Vincenza Colonna[1,2], Qasim Ayub[1], Yuan Chen[1], Luca Pagani[1,*], Pierre Luisi[3], Marc Pybus[3], Erik Garrison[4], Yali Xue[1],Chris Tyler-Smith[1], The 1000 Genomes Project Consortium[#]

[1]The Wellcome Trust Sanger Institute, Wellcome Trust Genome Campus, Hinxton, Cambs. CB10 1SA, UK
[2]Institute of Genetics and Biophysics 'A. Buzzati-Traverso', National Research Council (CNR), Naples, Italy
[3]Institute of Evolutionary Biology (Universitat Pompeu Fabra-CSIC), CEXS-UPF-PRBB, Barcelona, Catalonia, Spain.
[4]Department of Biology, Boston College, Chestnut Hill, Massachusetts, USA
[#] A full list of participants and institutions is available in the Supplement

*Present address: Laboratory of Molecular Anthropology, Department of Biological Geological and Environmental Sciences, University of Bologna, Bologna, Italy

This file includes Supplementary Figures 1 to 16

Supplementary Figure 1. Correlation between F_{ST} and ΔDAF a set of 5,000 random sites from chromosome 20

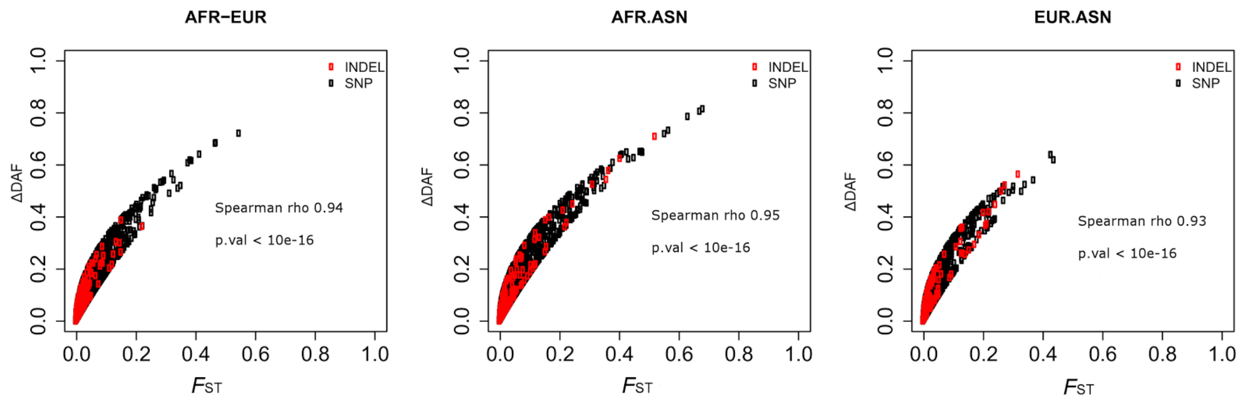

Supplementary Figure 2 . ΔDAF power to detect selection in simulated data. (See manuscript for details)

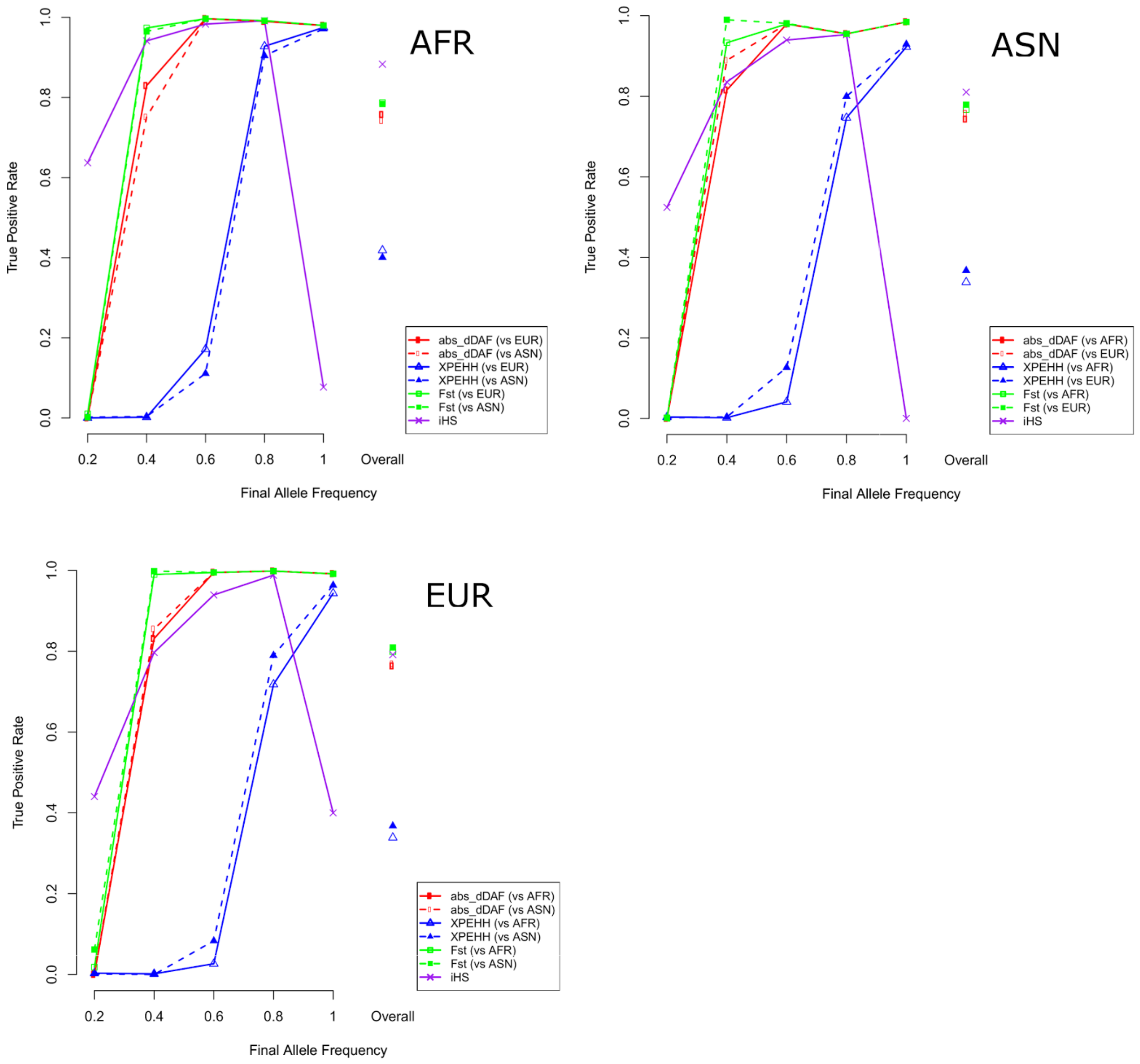

Supplementary Figure 3. Number of HighD sites according to different ΔDAF threshold and windows size.

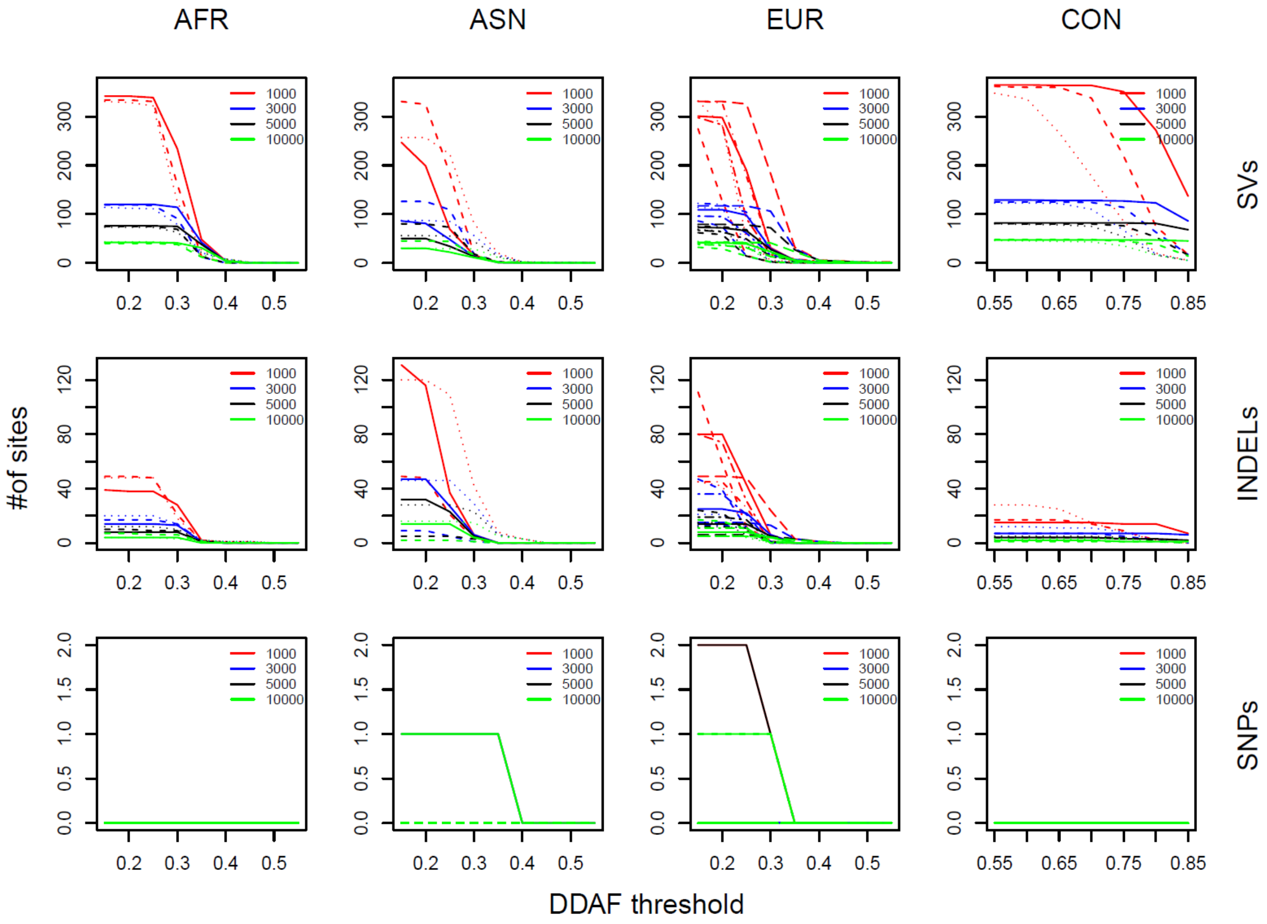

Supplementary Figure 4. Manhattan plots of rank p-values for cvDAFs at continental and populations levels. Chromosomes are represented by alternating turquoise and grey colors; red, blue and black dots represent INDEL, SNP and SV sites, respectively, that have been identified as low differentiated (LowD sites).

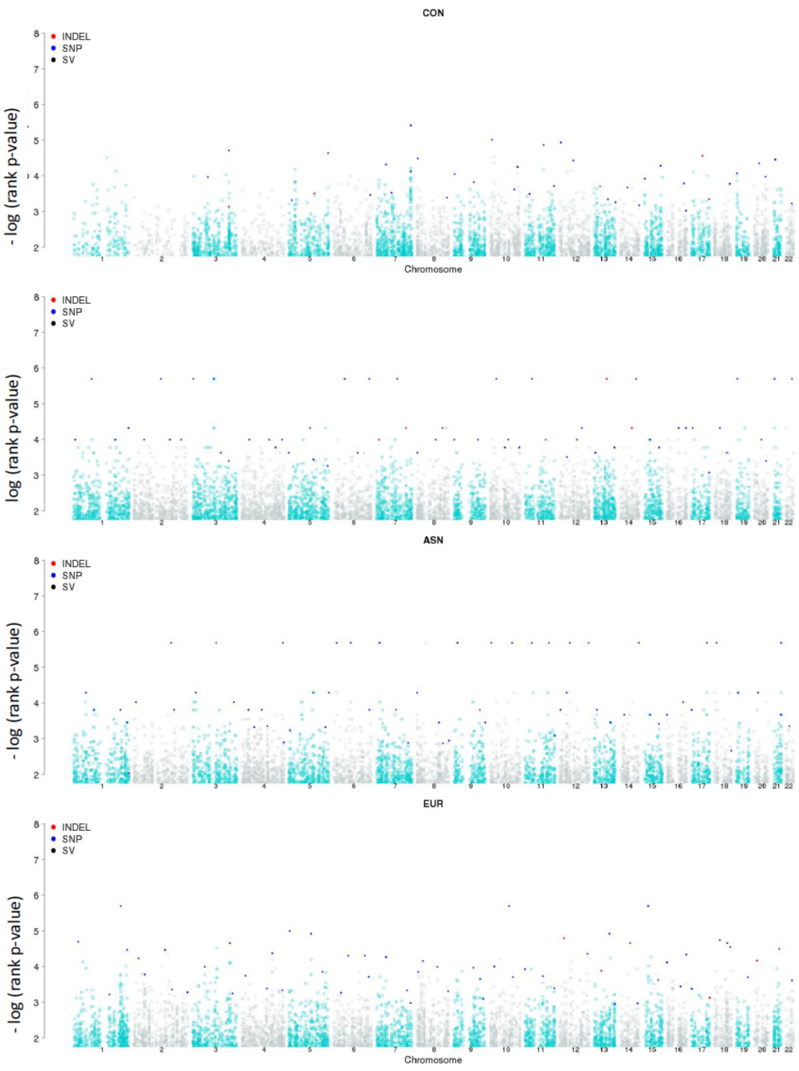

Supplementary Figure 5. Number of LowD sites according to different ΔDAF thresholds and windows sizes.

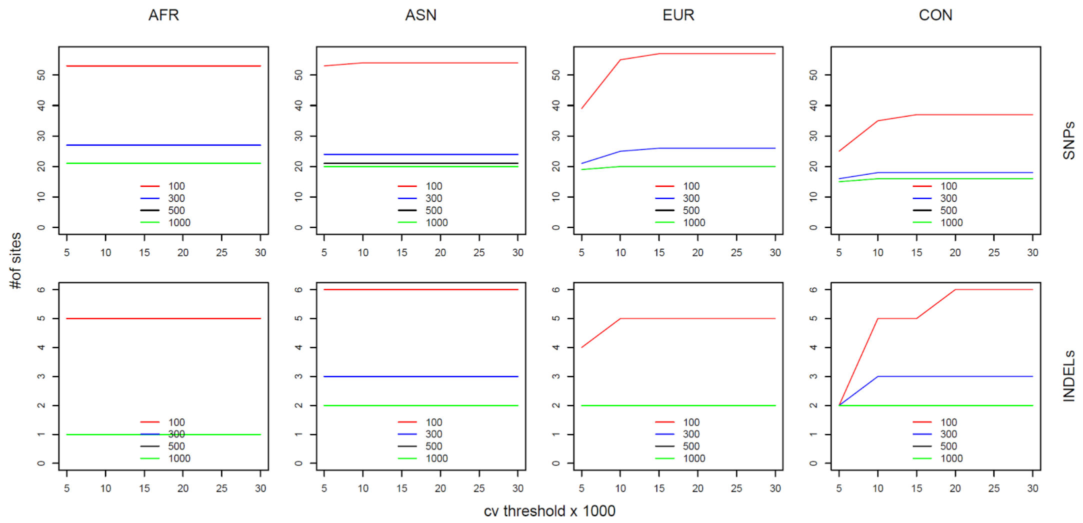

Supplementary Figure 6. Validation results in independent HapMap samples.

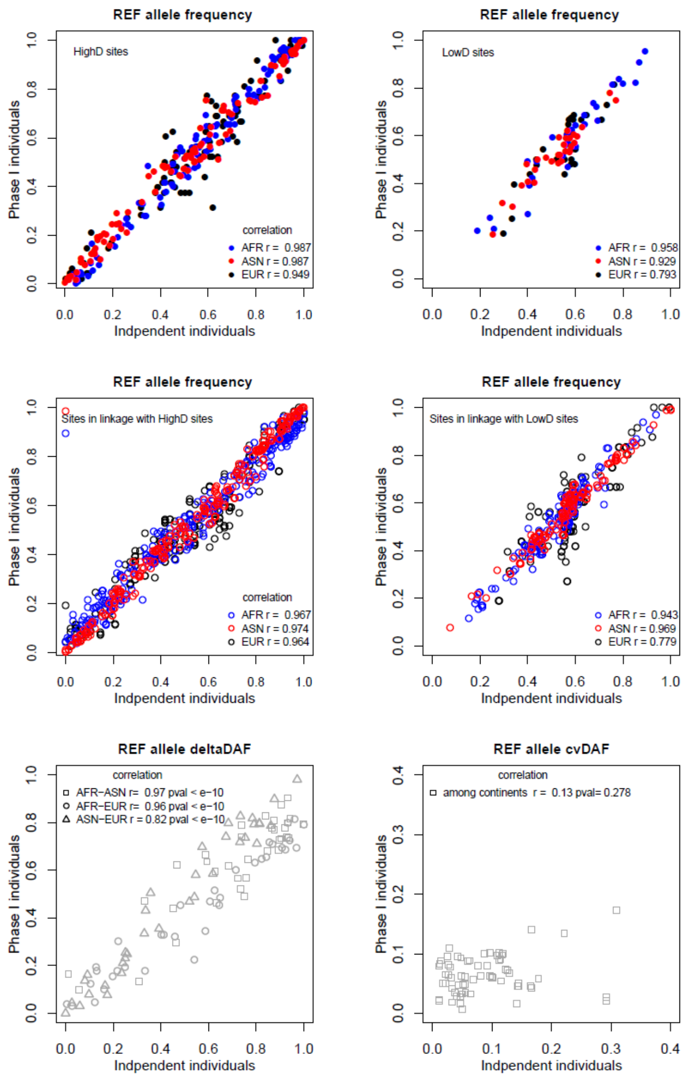

Supplementary Figure 7. Expected and observed number of HighD sites under different conditions of window sizes and ΔDAF thresholds.

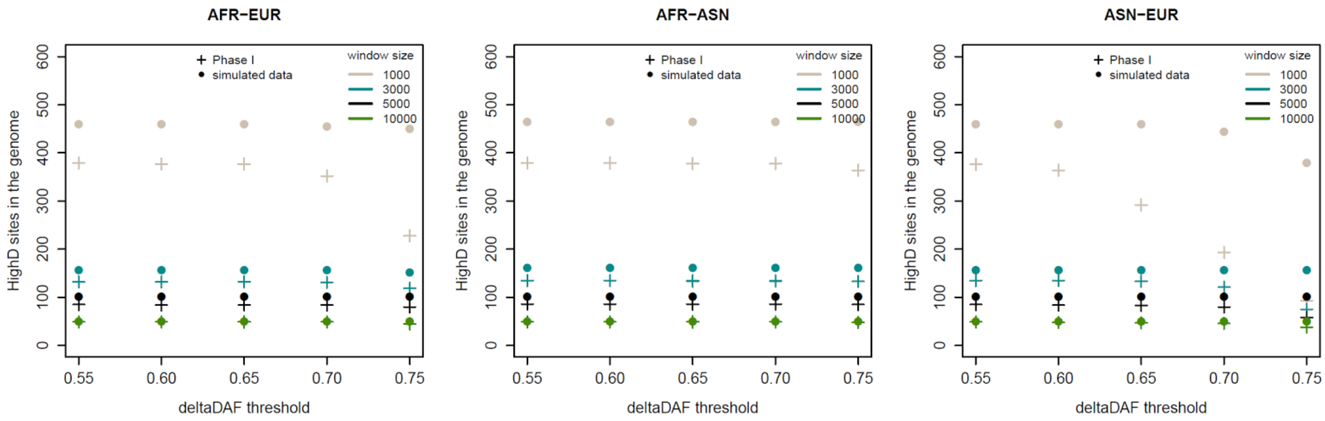

Supplementary Figure 8. Sensitivity to migration rate (m_rate) of the expected number of HighD sites from simulations under neutrality and comparison of two different demographic models. (a) Simulations of the AFR-EUR and ASN-EUR comparisons under the model proposed by Gravel and colleagues (Gravel S, et al. PNAS 2011, 108(29):11983-11988) as a complement to what presented in the main text. Dashed line represents number of observed HighD sites. (b) Comparison of allele frequency spectra of 1000 Genome data (red bars) with two published models (Gravel S, *et al.* PNAS 2011, 108(29):11983-11988 indicated as sims_g and Schaffner SF, *et al*. Genome Res 2005, 15(11):1576-1583 indicated as sims) and (c) number of expected HighD sites for simulated data under the model quoted above and another model (references X in the main text)

(a)

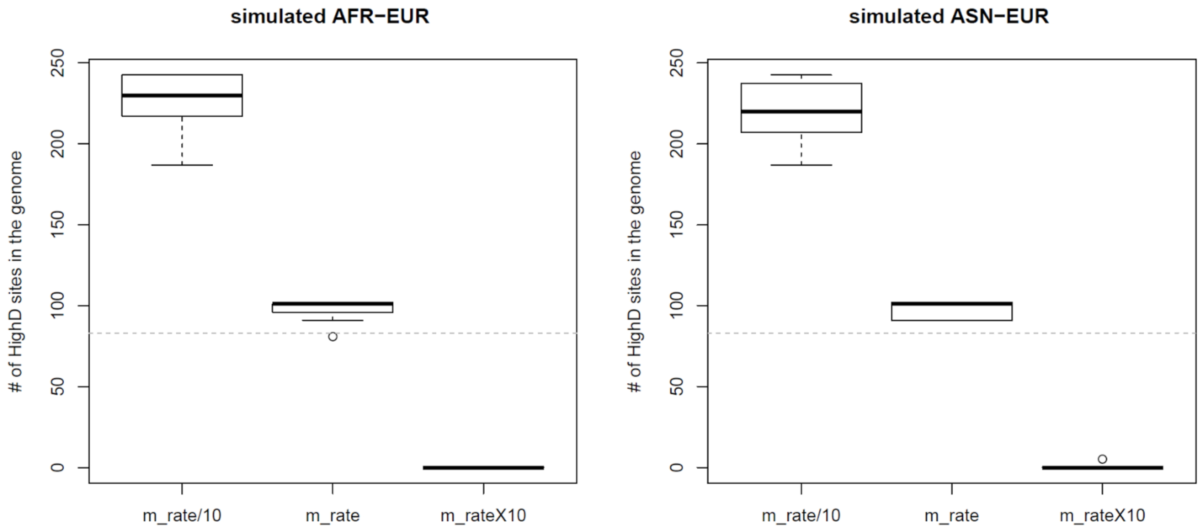

(b)

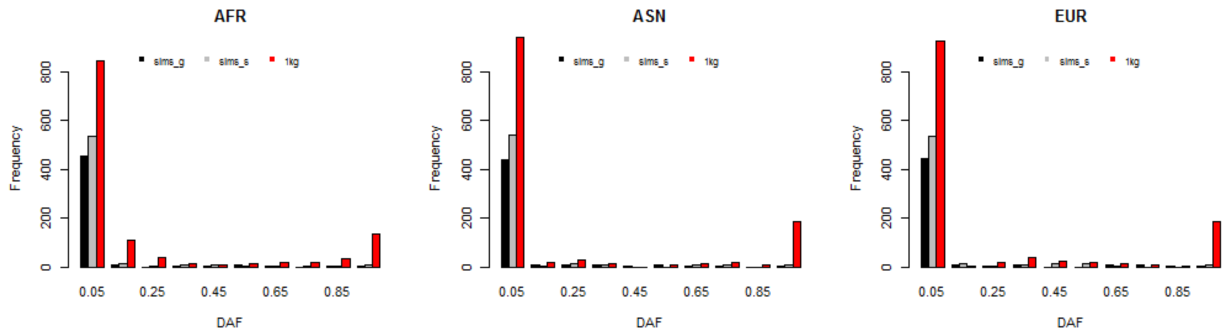
AFR
ASN
EUR
sims_g
sims_s
1kg
Frequency
0
200
400
600
800
0.05
0.25
0.45
0.65
0.85
DAF

(c)

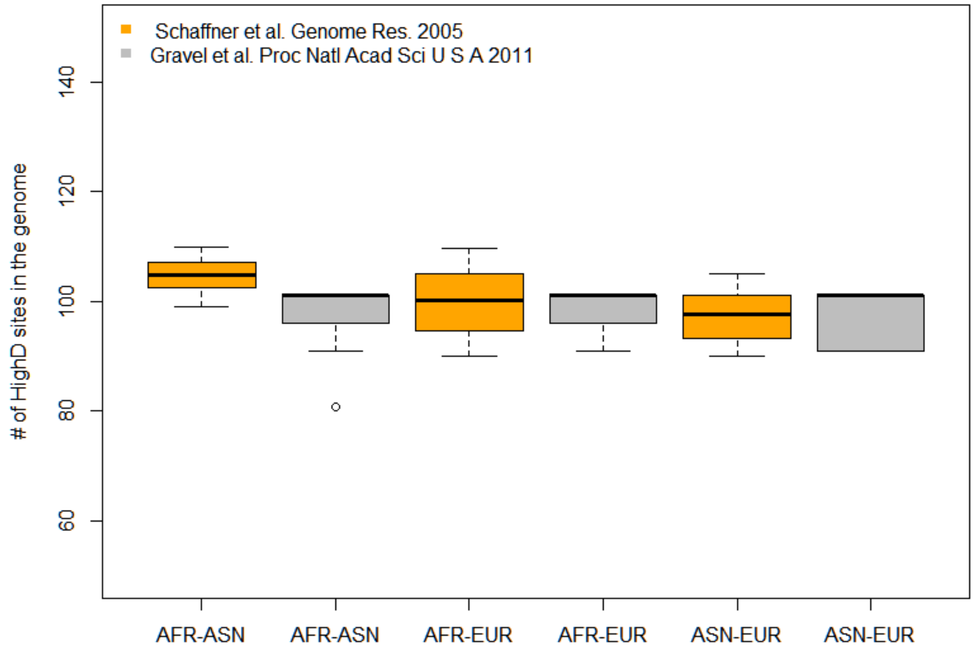
Schaffner et al. Genome Res. 2005
Gravel et al. Proc Natl Acad Sci U S A 2011
# of HighD sites in the genome
60
80
100
120
140
AFR-ASN
AFR-ASN
AFR-EUR
AFR-EUR
ASN-EUR
ASN-EUR

Supplementary Figure 9. Features of genomic sites matched for allele frequency and distance from gene.

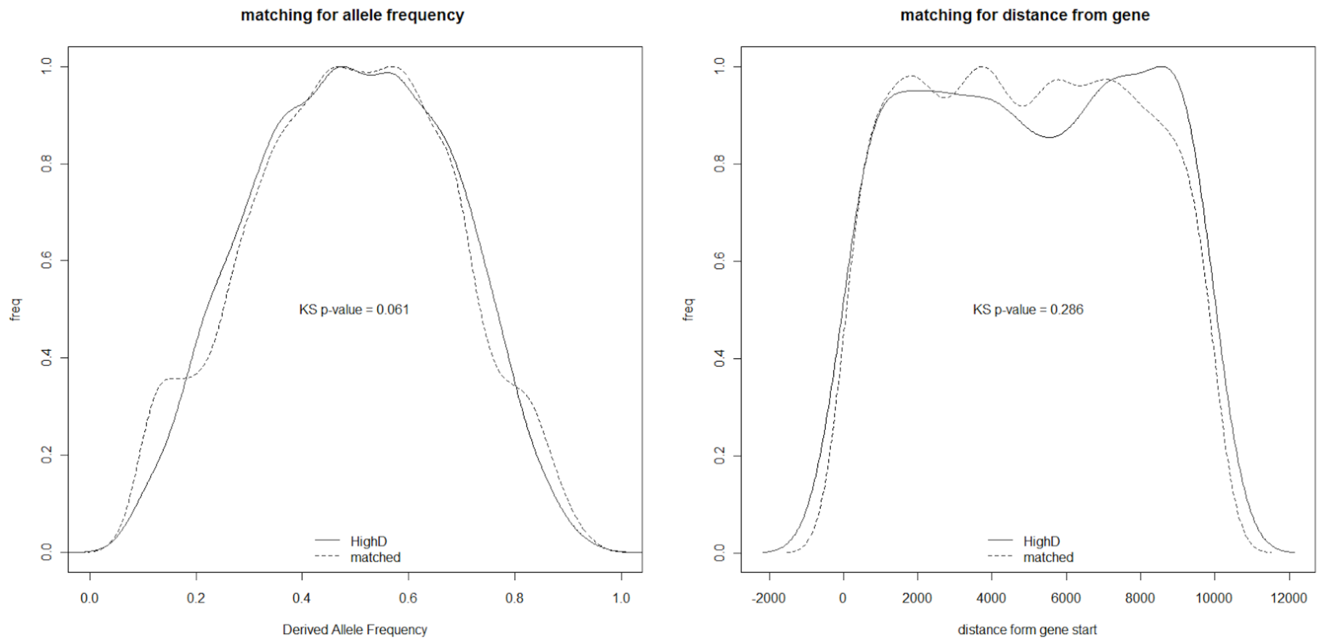

Supplementary Figure 10. Combined p-value for Tajima's D, Fay&Wu's H and Nielsen's CLR values in HighD sites and matched controls. Each column refer to a subset of HighD sites that have highest DAF in the population indicated in the head of the column; population indicated in rows are the population in which the combined p-values has been calculated for the set of sites in the column.

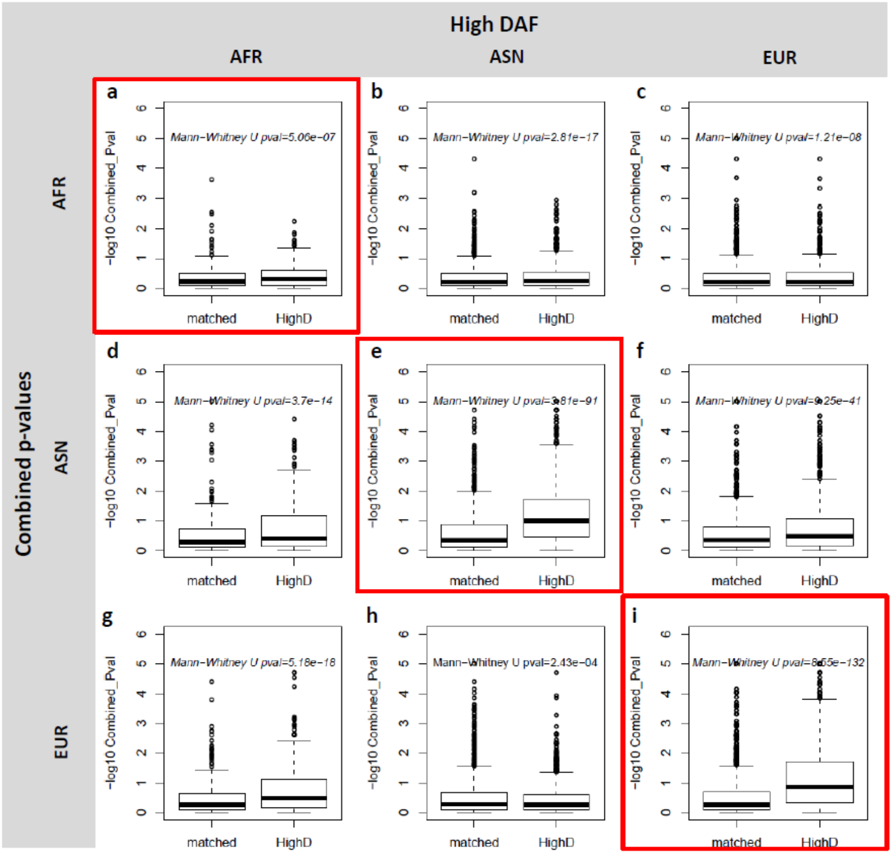

Supplementary Figure 11. Simulated data showing the frequency of the most common haplotype in 2kb surrounding a site under positive selection for a range of selective pressures leading to different final allele frequency at the selected site (on the x-axis). For hard sweeps (final allele frequency=1) there is mostly a single haplotype

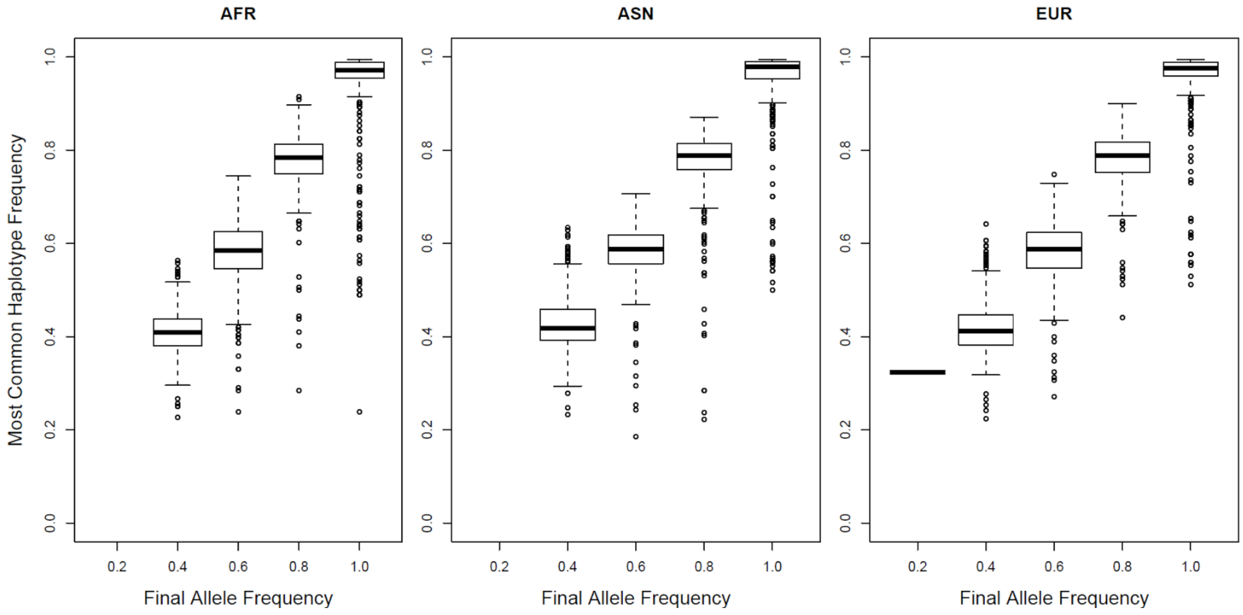

Supplementary Figure 12. Boxplots of Levenshtein distance from the major haplotype of all other haplotypes in 2kb surrounding sites under positive selection in simulated data. In the table below average values relative to boxplots. FaF=final allele frequency of the site under selection

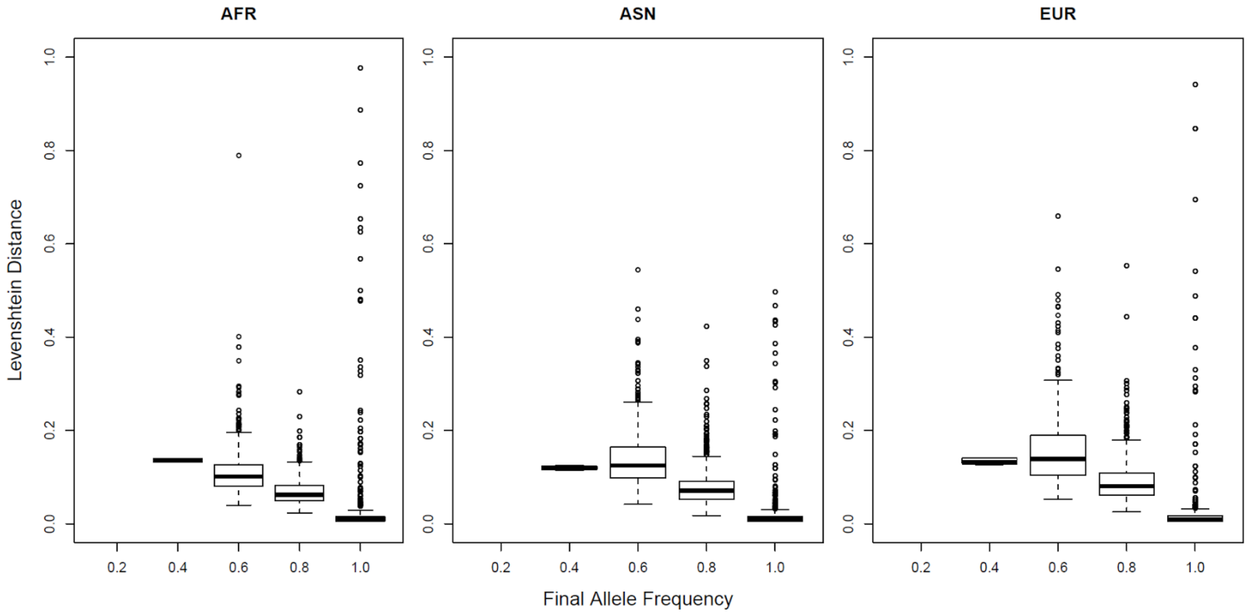

**FaF**	**EUR**	**ASN**	**AFR**
**0.4**	**0.1340252**	**0.1198131**	**0.1356534**
**0.6**	**0.1547858**	**0.1388615**	**0.1105228**
**0.8**	**0.09283899**	**0.08026323**	**0.06947882**
**1.0**	**0.03144997**	**0.02643067**	**0.04296775**

Supplementary Figure 13. Frequencies of haplotypes in 2 kb surrounding HighD sites demonstrated to be functional in examples of positive selection.

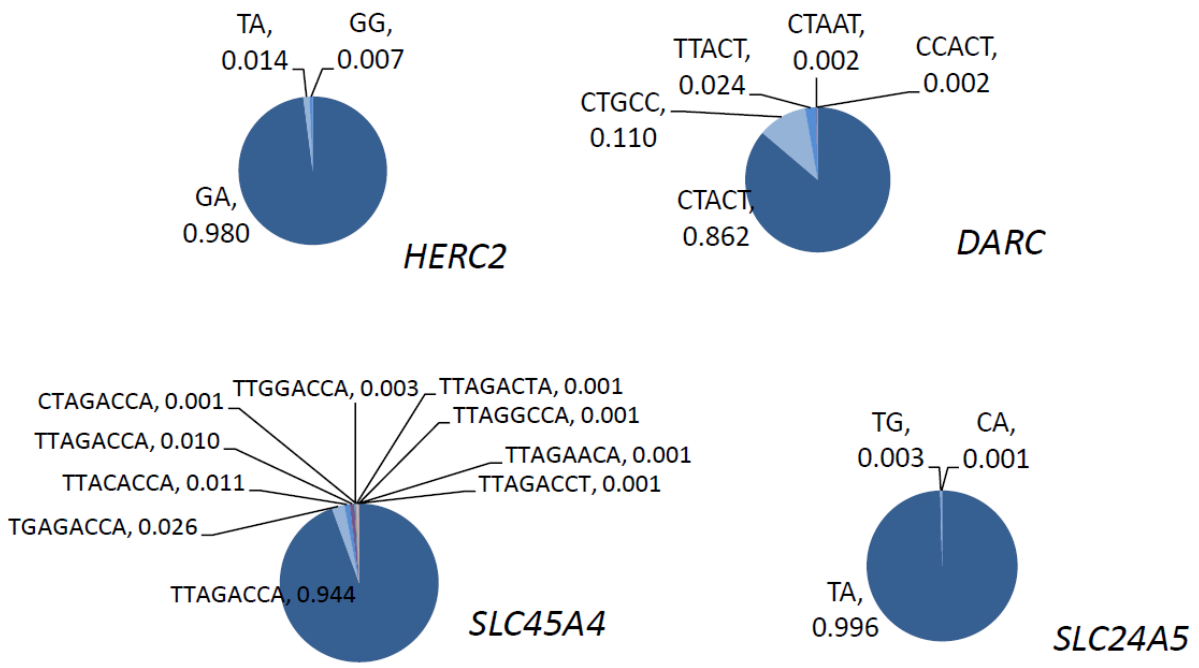

Supplementary Figure 14. Boxplots showing the distribution of weighted Levensthein distance in HighD sites and in matched controls for continental comparisons.

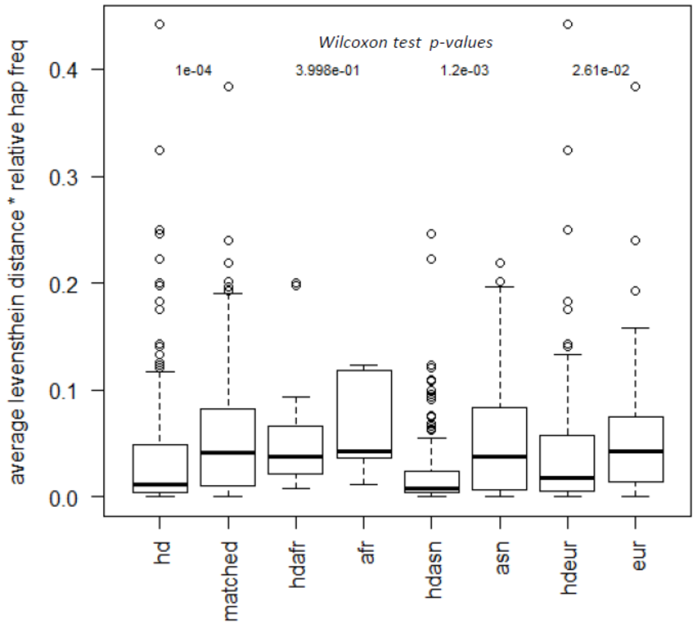

Supplementary Figure 15. Fraction of HighD and matched genomic sites with haplotypic features similar to sites accepted as examples of classic selective sweeps.

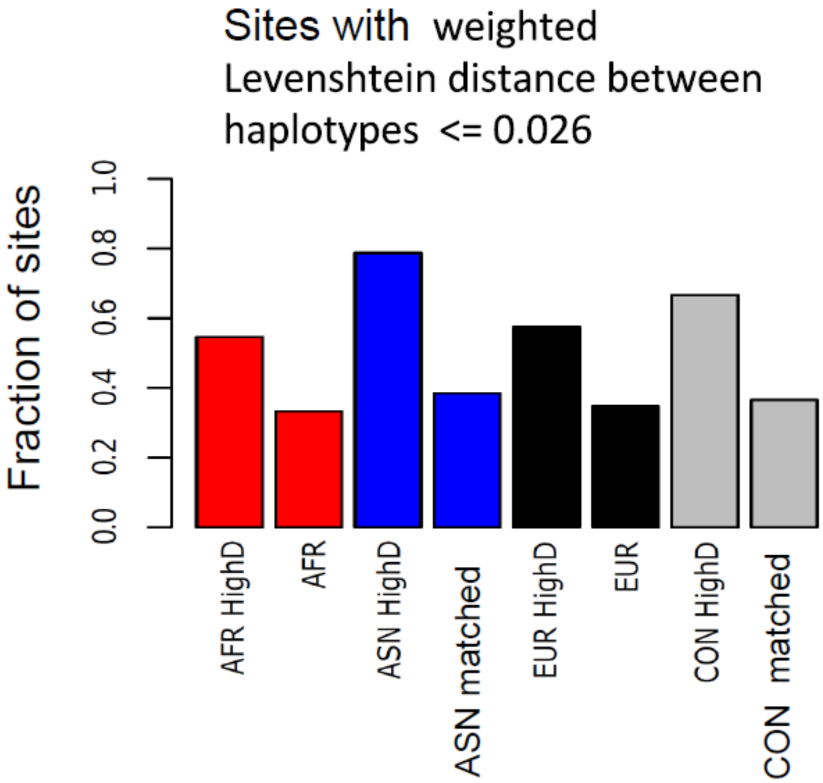

Supplementary Figure 16. Functional annotations in the genomic region surrounding the HighD site in *CALD1* and median joining network of haplotypes surrounding the site. Haplotypes are derived from sites in linkage disequilibrium (D’=1) with the HighD site in JPT populations.

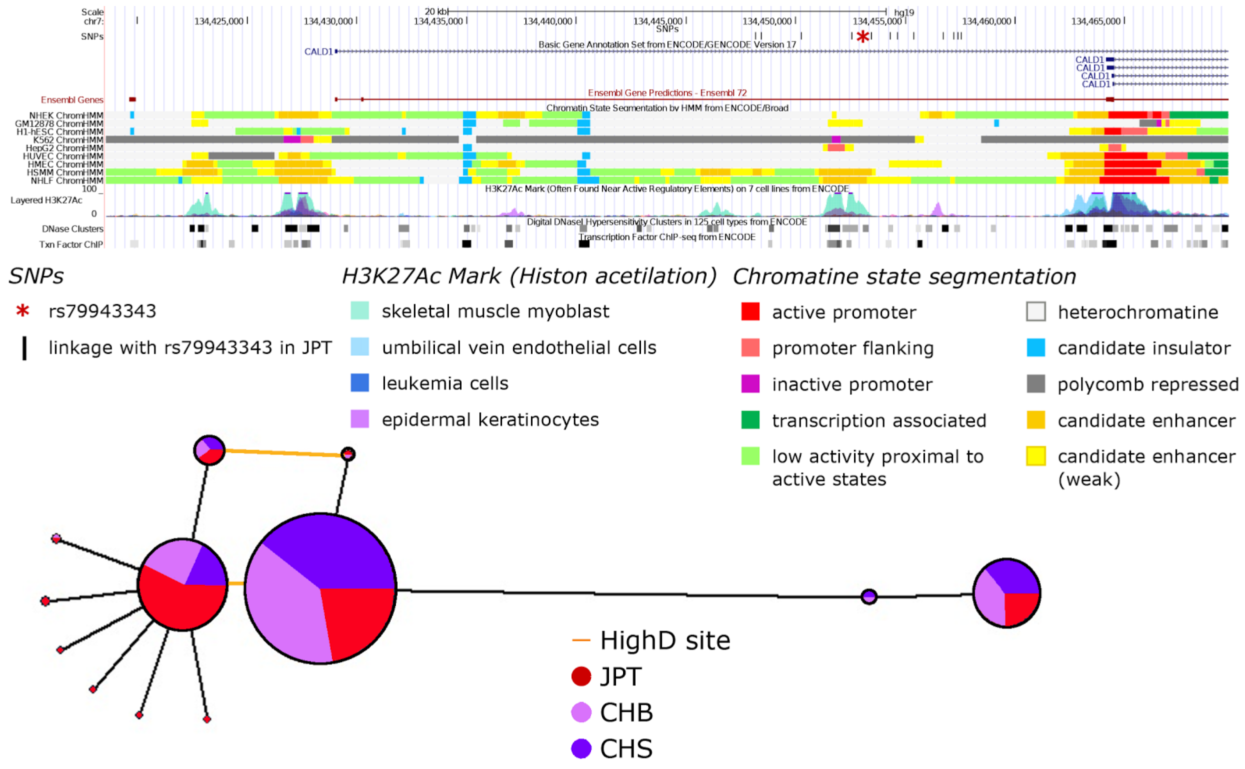

Supplement: Additional file 2 — This file contains supplementary Figures F1 to F16. [file gb-2014-15-6-r88-S2.pdf]
