# Supplementary material for: Human genomic regions with exceptionally high levels of population differentiation identified from 911 whole-genome sequences
Source: Genome Biol. 2014 Jun 30;15(6):R88. doi: 10.1186/gb-2014-15-6-r88 (PMC4197830; doi:10.1186/gb-2014-15-6-r88)
Supplement: Additional file 3 — Full list of participants and institutions in the 1000 Genomes Project. [file gb-2014-15-6-r88-S3.pdf]

The 1000 Genomes Consortium

Participants are arranged by project role, then by institution alphabetically, and finally alphabetically within institutions except for Principal Investigators and Project Leaders, as indicated.

Steering Committee: David M. Altshuler[3-5] (Co-Chair), Richard M. Durbin[6] (Co-Chair), Gonçalo R. Abecasis[7], David R. Bentley[8], Aravinda Chakravarti[9], Andrew G. Clark[10], Peter Donnelly[1,2], Evan E. Eichler[11], Paul Flicek[12], Stacey B. Gabriel[3], Richard A. Gibbs[13], Eric D. Green[14], Matthew E. Hurles[6], Bartha M. Knoppers[15], Jan O. Korbel[16], Eric S. Lander[3], Charles Lee[17], Hans Lehrach[18,27], Elaine R. Mardis[19], Gabor T. Marth[20], Gil A. McVean[1,2], Deborah A. Nickerson[21], Jeanette P. Schmidt[22], Stephen T. Sherry[23], Jun Wang[24], Richard K. Wilson[19]

Production Group: Baylor College of Medicine Richard A. Gibbs (Principal Investigator)[13], Huyen Dinh[13], Christie Kovar[13], Sandra Lee[13], Lora Lewis[13], Donna Muzny[13], Jeff Reid[13], Min Wang[13], **BGI-Shenzhen** Jun Wang (Principal Investigator)[24-26], Xiaodong Fang[24], Xiaosen Guo[24], Min Jian[24], Hui Jiang[24], Xin Jin[24], Guoqing Li[24], Jingxiang Li[24], Yingrui Li[24], Zhuo Li[24], Xiao Liu[24], Yao Lu[24], Xuedi Ma[24], Zhe Su[24],Shuaishuai Tai[24], Meifang Tang[24], Bo Wang[24], Guangbiao Wang[24], Honglong Wu[24], Renhua Wu[24], Ye Yin[24], Wenwei Zhang[24], Jiao Zhao[24], Meiru Zhao[24], Xiaole Zheng[24], Yan Zhou[24], **Broad Institute of MIT and Harvard** Eric S. Lander (Principal Investigator)[3], David M. Altshuler[3-5], Stacey B. Gabriel (Co-Chair)[3], Namrata Gupta[3], **European Bioinformatics Institute** Paul Flicek (Principal Investigator)[12], Laura Clarke[12], Rasko Leinonen[12], Richard E. Smith[12], Xiangqun Zheng-Bradley[12], **Illumina** David R. Bentley (Principal Investigator)[8], Russell Grocock[8], Sean Humphray[8], Terena James[8], Zoya Kingsbury[8], **Max Planck Institute for Molecular Genetics** Hans Lehrach (Principal Investigator)[18,27], Ralf Sudbrak (Project Leader)[18], Marcus W. Albrecht[28], Vyacheslav S. Amstislavskiy[18], Tatiana A. Borodina[28], Matthias Lienhard[18], Florian Mertes[18], Marc Sultan[18], Bernd Timmermann[18], Marie-Laure Yaspo[18], **US National Institutes of Health** Stephen T. Sherry (Principal Investigator)[23], **University of Oxford** Gil A. McVean (Principal Investigator)[1,2], **Washington University in St. Louis** Elaine R. Mardis (Co-Principal Investigator) (Co-Chair)[19], Richard K. Wilson (Co-Principal Investigator)[19], Lucinda Fulton[19], Robert Fulton[19], George M. Weinstock[19], **Wellcome Trust Sanger Institute** Richard M. Durbin (Principal Investigator)[6], Senduran Balasubramaniam[6], John Burton[6], Petr Danecek[6], Thomas M. Keane[6], Anja Kolb- Kokocinski[6], Shane McCarthy[6], James Stalker[6], Michael Quail[6]

Analysis Group: Affymetrix Jeanette P. Schmidt (Principal Investigator)[22], Christopher J. Davies[22], Jeremy Gollub[22], Teresa Webster[22], Brant Wong[22], Yiping Zhan[22], **Albert Einstein College of Medicine:** Adam Auton (Principal Investigator)[29], **Baylor College of Medicine** Richard A. Gibbs (Principal Investigator)[13], Fuli Yu (Project Leader)[13], Matthew Bainbridge[13], Danny Challis[13], Uday S. Evani[13], James Lu[13], Donna Muzny[13], Uma Nagaswamy[13], Jeff Reid[13], Aniko Sabo[13], Yi Wang[13], Jin Yu[13], **BGI-Shenzhen** Jun Wang (Principal Investigator)[24-26], Lachlan J.M. Coin[24], Lin Fang[24], Xiaosen Guo[24], Xin Jin[24], Guoqing Li[24], Qibin Li[24], Yingrui Li[24], Zhenyu Li[24], Haoxiang Lin[24], Binghang Liu[24], Ruibang Luo[24], Nan Qin[24], Haojing Shao[24], Bingqiang Wang[24], Yinlong Xie[24], Chen Ye[24], Chang Yu[24], Fan Zhang[24], Hancheng Zheng[24], Hongmei Zhu[24], **Boston College** Gabor T. Marth (Principal Investigator)[20], Erik P. Garrison[20], Deniz Kural[20], Wan-Ping Lee[20], Wen Fung Leong[20], Alistair N. Ward[20], Jiantao Wu[20], Mengyao Zhang[20], **Brigham and Women's Hospital** Charles Lee (Principal Investigator)[17], Lauren Griffin[17], Chih-Heng Hsieh[17], Ryan E. Mills[17,41],

Xinghua Shi[17], Marcin von Grotthuss[17], Chengsheng Zhang[17], **Broad Institute of MIT and Harvard** Mark J. Daly (Principal Investigator)[3], Mark A. DePristo (Project Leader)[3], David M. Altshuler[3-5], Eric Banks[3], Gaurav Bhatia[3], Mauricio O. Carneiro[3], Guillermo del Angel[3], Stacey B. Gabriel[3], Giulio Genovese[3], Namrata Gupta[3], Robert E. Handsaker[3,5], Chris Hartl[3], Eric S. Lander[3], Steven A. McCarroll[3], James C. Nemesh[3], Ryan E. Poplin[3], Stephen F. Schaffner[3], Khalid Shakir[3], **Cold Spring Harbor Laboratory** Seungtai C. Yoon (Principal Investigator)[30], Jayon Lihm[30],Vladimir Makarov[31], **Dankook University** Hanjun Jin (Principal Investigator)[32], Wook Kim[33], Ki Cheol Kim[33], **European Molecular Biology Laboratory** Jan O. Korbel (Principal Investigator)[16], Tobias Rausch[16], **European Bioinformatics Institute** Paul Flicek (Principal Investigator)[12], Kathryn Beal[12], Laura Clarke[12], Fiona Cunningham[12], Javier Herrero[12], William M. McLaren[12], Graham R.S. Ritchie[12], Richard E. Smith[12], Xiangqun Zheng-Bradley[12], **Cornell University** Andrew G. Clark (Principal Investigator)[10], Srikanth Gottipati[34], Alon Keinan[10], Juan L. Rodriguez-Flores[10], **Harvard University** Pardis C. Sabeti (Principal Investigator)[3,35], Sharon R. Grossman[3,35], Shervin Tabrizi[3,35], Ridhi Tariyal[3,35], **Human Gene Mutation Database** David N. Cooper (Principal Investigator)[36], Edward V. Ball[36], Peter D. Stenson[36], **Illumina** David R. Bentley (Principal Investigator)[8], Bret Barnes[37], Markus Bauer[8], R. Keira Cheetham[8], Tony Cox[8], Michael Eberle[8], Sean Humphray[8], Scott Kahn[37], Lisa Murray[8], John Peden[8], Richard Shaw[8], **Leiden University Medical Center** Kai Ye (Principal Investigator)[38], **Louisiana State University** Mark A. Batzer (Principal Investigator)[39], Miriam K. Konkel[39], Jerilyn A. Walker[39], **Massachusetts General Hospital** Daniel G. MacArthur (Principal Investigator)[40], Monkol Lek[40], **Max Planck Institute for Molecular Genetics** Ralf Sudbrak (Project Leader)[18], Vyacheslav S. Amstislavskiy[18], Ralf Herwig[18], **Pennsylvania State University** Mark D. Shriver (Principal Investigator)[42], **Stanford University** Carlos D. Bustamante (Principal Investigator)[43], Jake K. Byrnes[44], Francisco M. De La Vega[10], Simon Gravel[43], Eimear E. Kenny[43], Jeffrey M. Kidd[43], Phil Lacroute[43], Brian K. Maples[43], Andres Moreno-Estrada[43], Fouad Zakharia[43], **Tel-Aviv University** Eran Halperin (Principal Investigator)[45-47], Yael Baran[45], **Translational Genomics Research Institute** David W. Craig (Principal Investigator)[48], Alexis Christoforides[48], Nils Homer[110], Tyler Izatt[48], Ahmet A. Kurdoglu[48], Shripad A. Sinari[48], Kevin Squire[49], **US National Institutes of Health** Stephen T. Sherry (Principal Investigator)[23], Chunlin Xiao[23], **University of California, San Diego** Jonathan Sebat (Principal Investigator)[50,51], Vineet Bafna[52], Kenny Ye[53], **University of California, San Francisco** Esteban G. Burchard (Principal Investigator)[54], Ryan D. Hernandez (Principal Investigator)[54], Christopher R. Gignoux[54], **University of California, Santa Cruz** David Haussler (Principal Investigator)[55,111], Sol J. Katzman[55], W. James Kent[55], **University of Chicago** Bryan Howie[56], **University College London** Andres Ruiz-Linares (Principal Investigator)[57], **University of Geneva** Emmanouil T. Dermitzakis (Principal Investigator)[58,59,104], Tuuli Lappalainen[58,59,104], **University of Maryland School of Medicine** Scott E. Devine (Principal Investigator)[60], Xinyue Liu[60], Ankit Maroo[60], Luke J. Tallon[60], **University of Medicine and Dentistry of New Jersey** Jeffrey A. Rosenfeld (Principal Investigator)[61,62], Leslie P. Michelson[61], **University of Michigan** Gonçalo R. Abecasis (Principal Investigator) (Co-Chair)[7], Hyun Min Kang (Project Leader)[7], Paul Anderson[7], Andrea Angius[106], Abigail Bigham[63], Tom Blackwell[7], Fabio Busonero[7,105,106], Francesco Cucca[105,106], Christian Fuchsberger[7], Chris Jones[107], Goo Jun[7], Yun Li[64], Robert Lyons[108], Andrea Maschio[7,105,106], Eleonora Porcu[7,105,106], Fred Reinier[107], Serena Sanna[106], David Schlessinger[109], Carlo Sidore[7,105,106], Adrian Tan[7], Mary Kate Trost[7], **University of Montréal** Philip Awadalla (Principal Investigator)[65], Alan Hodgkinson[65], **University of Oxford** Gerton Lunter (Principal Investigator)[1], Gil A. McVean (Principal Investigator) (Co-Chair)[1,2], Jonathan L. Marchini (Principal Investigator)[1,2], Simon Myers (Principal Investigator)[1,2], Claire Churchhouse[2], Olivier Delaneau[2], Anjali Gupta-Hinch[1], Zamin Iqbal[1], Iain Mathieson[1], Andy Rimmer[1], Dionysia K. Xifara[1,2], **University of Puerto Rico** Taras K. Oleksyk (Principal Investigator)[66], **University of Texas Health Sciences Center at Houston** Yunxin Fu (Principal Investigator)[67], Xiaoming Liu[67],Momiao Xiong[67], **University of Utah** Lynn Jorde (Principal Investigator)[68], David Witherspoon[68], Jinchuan Xing[69], **University of Washington** Evan E. Eichler (Principal Investigator)[11], Brian L. Browning (Principal Investigator)[70], Can Alkan[21,71],

Iman Hajirasouliha[102], Fereydoun Hormozdiari[21], Arthur Ko[21], Peter H. Sudmant[21] **Washington University in St. Louis** Elaine R. Mardis (Co-Principal Investigator)[19], Ken Chen[103], Asif Chinwalla[19], Li Ding[19], David Dooling[19], Daniel C. Koboldt[19], Michael D. McLellan[19], John W. Wallis[19], Michael C. Wendl[19], Qunyuan Zhang[19], **Wellcome Trust Sanger Institute** Richard M. Durbin (Principal Investigator)[6], Matthew E. Hurles (Principal Investigator)[6], Chris Tyler-Smith (Principal Investigator)[6], Cornelis A. Albers[72], Qasim Ayub[6], Senduran Balasubramaniam[6], Yuan Chen[6], Alison J. Coffey[6], Vincenza Colonna[6,73], Petr Danecek[6], Ni Huang[6], Luke Jostins[6], Thomas M. Keane[6], Heng Li[3,6], Shane McCarthy[6], Aylwyn Scally[6], James Stalker[6], Klaudia Walter[6], Yali Xue[6], Yujun Zhang[6], **Yale University** Mark B. Gerstein (Principal Investigator)[74-76], Alexej Abyzov[74, 76], Suganthi Balasubramanian[76], Jieming Chen[74], Declan Clarke[77], Yao Fu[74], Lukas Habegger[74], Arif O. Harmanci[74], Mike Jin[76], Ekta Khurana[76], Xinmeng Jasmine Mu[74], Cristina Sisu[74]

Structural Variation Group: BGI-Shenzhen Yingrui Li[24], Ruibang Luo[24], Hongmei Zhu[24], **Brigham and Women's Hospital** Charles Lee (Principal Investigator) (Co-Chair)[17], Lauren Griffin[17], Chih-Heng Hsieh[17], Ryan E. Mills[17,41], Xinghua Shi[17], Marcin von Grotthuss[17], Chengsheng Zhang[17], **Boston College** Gabor T. Marth (Principal Investigator)[20], Erik P. Garrison[20], Deniz Kural[20], Wan-Ping Lee[20], Alistair N. Ward[20], Jiantao Wu[20], Mengyao Zhang[20], **Broad Institute of MIT and Harvard** Steven A. McCarroll (Project Lead)[3], David M. Altshuler[3-5], Eric Banks[3], Guillermo del Angel[3], Giulio Genovese[3], Robert E. Handsaker[3,5], Chris Hartl[3], James C. Nemesh[3], Khalid Shakir[3], **Cold Spring Harbor Laboratory** Seungtai C. Yoon (Principal Investigator)[30], Jayon Lihm[30], Vladimir Makarov[31], **Cornell University** Jeremiah Degenhardt[10], **European Bioinformatics Institute** Paul Flicek (Principal Investigator)[12], Laura Clarke[12], Richard E. Smith[12], Xiangqun Zheng-Bradley[12], **European Molecular Biology Laboratory** Jan O. Korbel (Principal Investigator) (Co-Chair)[16], Tobias Rausch[16], Adrian M. Stütz[16], **Illumina** David R. Bentley (Principal Investigator)[8], Bret Barnes[37], R. Keira Cheetham[8], Michael Eberle[8], Sean Humphray[8], Scott Kahn[37], Lisa Murray[8], Richard Shaw[8], **Leiden University Medical Center** Kai Ye (Principal Investigator)[38], **Louisiana State University** Mark A. Batzer (Principal Investigator)[39], Miriam K. Konkel[39], Jerilyn A. Walker[39], **Stanford University** Phil Lacroute[43], **Translational Genomics Research Institute** David W. Craig (Principal Investigator)[48], Nils Homer[110], **US National Institutes of Health** Deanna Church[23], Chunlin Xiao[23], **University of California, San Diego** Jonathan Sebat (Principal Investigator)[50,51], Vineet Bafna[52], Jacob J. Michaelson[79], Kenny Ye[53], **University of Maryland School of Medicine** Scott E. Devine (Principal Investigator)[60], Xinyue Liu[60], Ankit Maroo[60], Luke J. Tallon[60], **University of Oxford** Gerton Lunter (Principal Investigator)[1], Gil A. McVean (Principal Investigator)[1,2], Zamin Iqbal[1], **University of Utah** David Witherspoon[68], Jinchuan Xing[69], **University of Washington** Evan E. Eichler (Principal Investigator) (Co-Chair)[11], Can Alkan[21,71], Iman Hajirasouliha[102], Fereydoun Hormozdiari[21], Arthur Ko[21], Peter H. Sudmant[21], **Washington University in St. Louis** Ken Chen[103], Asif Chinwalla[19], Li Ding[19], Michael D. McLellan[19], John W. Wallis[19], **Wellcome Trust Sanger Institute** Matthew E. Hurles (Principal Investigator) (Co-Chair)[6], Ben Blackburne[6], Heng Li[6], Sarah J. Lindsay[6], Zemin Ning[6], Aylwyn Scally[6], Klaudia Walter[6], Yujun Zhang[6], **Yale University** Mark B. Gerstein (Principal Investigator)[74-76], Alexej Abyzov[74,76], Jieming Chen[74], Declan Clarke[77], Ekta Khurana[76], Xinmeng Jasmine Mu[74], Cristina Sisu[74]

Exome Group: Baylor College of Medicine Richard A. Gibbs (Principal Investigator) (Co-Chair)[13], Fuli Yu (Project Leader)[13], Matthew Bainbridge[13], Danny Challis[13], Uday S.Evani[13], Christie Kovar[13], Lora Lewis[13], James Lu[13], Donna Muzny[13], Uma Nagaswamy[13], Jeff Reid[13], Aniko Sabo[13], Jin Yu[13], **BGI-Shenzhen** Xiaosen Guo[24],Yingrui Li[24], Renhua Wu[24], **Boston College** Gabor T. Marth (Principal Investigator) (Co-Chair)[20], Erik P. Garrison[20], Wen Fung Leong[20], Alistair N. Ward[20], **Broad Institute of MIT and Harvard** Guillermo del Angel[3], Mark A. DePristo[3], Stacey B. Gabriel[3], Namrata Gupta[3], Chris Hartl[3], Ryan E. Poplin[3], **Cornell**

University Andrew G. Clark (Principal Investigator)[10], Juan L. Rodriguez-Flores[10], **European Bioinformatics Institute** Paul Flicek (Principal Investigator)[12], Laura Clarke[12], Richard E. Smith[12], Xiangqun Zheng-Bradley[12], **Massachusetts General Hospital** Daniel G. MacArthur (Principal Investigator)[40], **Stanford University** Carlos D. Bustamante (Principal Investigator)[43], Simon Gravel[43], **Translational Genomics Research Institute** David W. Craig (Principal Investigator)[48], Alexis Christoforides[48], Nils Homer[110], Tyler Izatt[48], **US National Institutes of Health** Stephen T. Sherry (Principal Investigator)[23], Chunlin Xiao[23], **University of Geneva** Emmanouil T. Dermitzakis (Principal Investigator)[58,59,104], **University of Michigan** Gonçalo R. Abecasis (Principal Investigator)[7], Hyun Min Kang[7], **University of Oxford** Gil A. McVean (Principal Investigator)[1,2], **Washington University in St. Louis** Elaine R. Mardis (Principal Investigator)[19], David Dooling[19], Lucinda Fulton[19], Robert Fulton[19], Daniel C. Koboldt[19], **Wellcome Trust Sanger Institute** Richard M. Durbin (Principal Investigator)[6], Senduran Balasubramaniam[6], Thomas M. Keane[6], Shane McCarthy[6], James Stalker[6], **Yale University** Mark B. Gerstein (Principal Investigator)[74-76], Suganthi Balasubramanian[76], Lukas Habegger[74]

Functional Interpretation Group: Boston College Erik P. Garrison[20], **Baylor College of Medicine** Richard A. Gibbs (Principal Investigator) [13], Matthew Bainbridge[13], Donna Muzny[13], Fuli Yu[13], Jin Yu[13], **Broad Institute of MIT and Harvard** Guillermo del Angel[3], Robert E. Handsaker[3,5], **Cold Spring Harbor Laboratory** Vladimir Makarov[31], **Cornell University** Juan L. Rodriguez-Flores[10], **Dankook University** Hanjun Jin (Principal Investigator)[32], Wook Kim[33], Ki Cheol Kim[33], **European Bioinformatics Institute** Paul Flicek (Principal Investigator)[12], Kathryn Beal[12], Laura Clarke[12], Fiona Cunningham[12], Javier Herrero[12], William M. McLaren[12], Graham R.S. Ritchie[12], Xiangqun Zheng-Bradley[12], **Harvard University** Shervin Tabrizi[3,35], **Massachusetts General Hospital** Daniel G. MacArthur (Principal Investigator)[40], Monkol Lek[40], **Stanford University** Carlos D. Bustamante (Principal Investigator)[43], Francisco M. De La Vega[10], **Translational Genomics Research Institute** David W. Craig (Principal Investigator)[48],Ahmet A. Kurdoglu[48], **University of Geneva** Tuuli Lappalainen[58,59,104], **University of Medicine and Dentistry of New Jersey** Jeffrey A. Rosenfeld (Principal Investigator)[61,62], Leslie P. Michelson[61,62], **University of Montréal** Philip Awadalla (Principal Investigator)[65], Alan Hodgkinson[65], **University of Oxford** Gil A. McVean (Principal Investigator)[1,2], **Washington University in St. Louis** Ken Chen[103], **Wellcome Trust Sanger Institute** Chris Tyler-Smith (Principal Investigator) (Co-Chair)[6], YuanChen[6], Vincenza Colonna[6,73], Adam Frankish[6], Jennifer Harrow[6], Yali Xue[6], **Yale University** Mark B. Gerstein (Principal Investigator) (Co-Chair)[74-76], Alexej Abyzov[74,76], Suganthi Balasubramanian[76], Jieming Chen[74], Declan Clarke[77], Yao Fu[74], Arif O. Harmanci[74], Mike Jin[76], Ekta Khurana[76], Xinmeng Jasmine Mu[74], Cristina Sisu[74]

Data Coordination Center Group: Baylor College of Medicine Richard A. Gibbs (Principal Investigator)[13], Christie Kovar[13], Divya Kalra[13], Walker Hale[13], Gerald Fowler[13], Donna Muzny[13], Jeff Reid[13], **BGI-Shenzhen** Jun Wang (Principal Investigator)[24,26], Xiaosen Guo[24], Guoqing Li[24], Yingrui Li[24], Xiaole Zheng[24], **Broad Institute of MIT and Harvard** David M. Altshuler[3-5], **European Bioinformatics Institute** Paul Flicek (Principal Investigator) (Co-Chair)[12], Laura Clarke (Project Lead)[12], Jonathan Barker[12], Gavin Kelman[12], Eugene Kulesha[12], Rasko Leinonen[12], William M. McLaren[12], Rajesh Radhakrishnan[12], Asier Roa[12], Dmitriy Smirnov[12], Richard E. Smith[12], Ian Streeter[12], Iliana Toneva[12], Brendan Vaughan[12], Xiangqun Zheng-Bradley[12], **Illumina** David R. Bentley (Principal Investigator)[8], Tony Cox[8], Sean Humphray[8], Scott Kahn[37], **Max Planck Institute for Molecular Genetics** Ralf Sudbrak (Project Lead)[18], Marcus W. Albrecht[28], Matthias Lienhard[18], **Translational Genomics Research Institute** David W. Craig (Principal Investigator)[48], Tyler Izatt[48], Ahmet A. Kurdoglu[48], **US National Institutes of Health** Stephen T. Sherry (Principal Investigator) (Co-Chair)[23], Victor Ananiev[23], Zinaida Belaia[23], Dimitriy Beloslyudtsev[23], Nathan Bouk[23], Chao Chen[23],Deanna Church[23], Robert Cohen[23], Charles

Cook[23], John Garner[23], Timothy Hefferon[23], Mikhail Kimelman[23], Chunlei Liu[23], John Lopez[23], Peter Meric[23], Chris O'Sullivan[80], Yuri Ostapchuk[23], Lon Phan[23], Sergiy Ponomarov[23], Valerie Schneider[23], Eugene Shekhtman[23], Karl Sirotkin[23], Douglas Slotta[23], Chunlin Xiao[23], Hua Zhang[23], **University of California, Santa Cruz** David Haussler (Principal Investigator)[55,111], **University of Michigan** Gonçalo R. Abecasis (Principal Investigator)[7], **University of Oxford** Gil A. McVean (Principal Investigator)[1,2], **University of Washington** Can Alkan[21,71], Arthur Ko[21], **Washington University in St. Louis** David Dooling[19], **Wellcome Trust Sanger Institute** Richard M. Durbin (Principal Investigator)[6], Senduran Balasubramaniam[6], Thomas M. Keane[6], Shane McCarthy[6], James Stalker[6]

Samples and ELSI Group: Aravinda Chakravarti (Co-Chair)[9], Bartha M. Knoppers (Co-Chair)[15], Gonçalo R. Abecasis[7], Kathleen C. Barnes[81], Christine Beiswanger[82], Esteban Burchard[54], Carlos D. Bustamante[43], Hongyu Cai[24], Hongzhi Cao[24], Richard M. Durbin[6], Neda Gharani[82], Richard A. Gibbs[13], Christopher R. Gignoux[54], Simon Gravel[43], Brenna Henn[43], Danielle Jones[34], Lynn Jorde[68], Jane S. Kaye[83], Alon Keinan[10], Alastair Kent[84], Angeliki Kerasidou[1], Yingrui Li[24], Rasika Mathias[85], Gil McVean[1,2], Andres Moreno-Estrada[43], Pilar N. Ossorio[86,87], Michael Parker[88], David Reich[5], Charles N. Rotimi[89], Charmaine D. Royal[90], Karla Sandoval[43], Yeyang Su[24], Ralf Sudbrak[18], Zhongming Tian[24], Bernd Timmermann[18], Sarah Tishkoff[91], Lorraine H. Toji[82], Chris Tyler-Smith[6], Marc Via[92], Yuhong Wang[24], Huanming Yang[24], Ling Yang[24], Jiayong Zhu[24]

Sample Collection: British from England and Scotland (GBR) Walter Bodmer[93], **Colombians in Medellín, Colombia (CLM)** Gabriel Bedoya[94], Andres Ruiz-Linares[57], **Han Chinese South (CHS)** Cai Zhi Ming[24], Gao Yang[95], Chu Jia You[96], **Finnish in Finland (FIN)** Leena Peltonen[‡], **Iberian Populations in Spain (IBS)** Andres Garcia-Montero[97], Alberto Orfao[98], **Puerto Ricans in Puerto Rico (PUR)** Julie Dutil[99], Juan C. Martinez-Cruzado[66], Taras K. Oleksyk[66]

Scientific Management: Lisa D. Brooks[100], Adam L. Felsenfeld[100], Jean E. McEwen[100], Nicholas C. Clemm[100], Audrey Duncanson[101], Michael Dunn[101], Eric D. Green[14], Mark S. Guyer[100], Jane L. Peterson[100]

Writing Group: Goncalo R. Abecasis[7], Adam Auton[29], Lisa D. Brooks[100], Mark A. DePristo[3], Richard M. Durbin[6], Robert E. Handsaker[3,5], Hyun Min Kang[7], Gabor T. Marth[20], Gil A. McVean[1,2]

1 Wellcome Trust Centre for Human Genetics, Oxford University, Oxford OX3 7BN, UK.

2 Dept of Statistics, Oxford University, Oxford OX1 3TG, UK.

3 The Broad Institute of MIT and Harvard, 7 Cambridge Center, Cambridge,Massachusetts 02142, USA.

4 Center for Human Genetic Research, Massachusetts General Hospital, Boston, Massachusetts 02114, USA.

5 Dept of Genetics, Harvard Medical School, Cambridge, Massachusetts 02142, USA.

6 Wellcome Trust Sanger Institute, Wellcome Trust Genome Campus, Cambridge,CB10 1SA, UK.

7 Center for Statistical Genetics, Biostatistics, University of Michigan, Ann Arbor, Michigan 48109, USA.

8 Illumina United Kingdom, Chesterford Research Park, Little Chesterford, Nr Saffron Walden, Essex CB10 1XL, UK.

9 McKusick-Nathans Institute of Genetic Medicine, Johns Hopkins University School of Medicine, Baltimore, Maryland 21205, USA.

10 Center for Comparative and Population Genomics, Cornell University, Ithaca, New York 14850, USA.

11 Dept of Genome Sciences, University of Washington School of Medicine and Howard Hughes Medical Institute, Seattle, Washington 98195, USA.

12 European Bioinformatics Institute, Wellcome Trust Genome Campus, Cambridge, CB10 1SD, UK.

13 Baylor College of Medicine, Human Genome Sequencing Center, Houston, Texas 77030, USA.

14 US National Institutes of Health, National Human Genome Research Institute, 31 Center Drive, Bethesda, Maryland 20892, USA.

15 Centre of Genomics and Policy, McGill University, Montréal, Québec H3A 1A4, Canada.

16 European Molecular Biology Laboratory, Genome Biology Research Unit, Meyerhofstraße 1, 69117 Heidelberg, Germany.

17 Dept of Pathology, Brigham and Women's Hospital and Harvard Medical School, Boston, Massachusetts 02115, USA.

18 Max Planck Institute for Molecular Genetics, Ihnestraße 63-73, 14195 Berlin, Germany.

19 The Genome Center, Washington University School of Medicine, St Louis, Missouri 63108, USA.

20 Dept of Biology, Boston College, Chestnut Hill, Massachusetts 02467, USA.

21 Dept of Genome Sciences, University of Washington School of Medicine, Seattle, Washington 98195, USA.

22 Affymetrix, Inc., Santa Clara, California 95051, USA.

23 US National Institutes of Health, National Center for Biotechnology Information, 45 Center Drive, Bethesda, Maryland 20892, USA.

24 BGI-Shenzhen, Shenzhen 518083, China.

25 The Novo Nordisk Foundation Center for Basic Metabolic Research, University of Copenhagen, DK-2200 Copenhagen, Denmark.

26 Dept of Biology, University of Copenhagen, DK-2100 Copenhagen, Denmark.

27 Dahlem Centre for Genome Research and Medical Systems Biology, D-14195 Berlin-Dahlem, Germany.

28 Alacris Theranostics GmbH, D-14195 Berlin-Dahlem, Germany.

29 Dept of Genetics, Albert Einstein College of Medicine, Bronx, New York 10461, USA.

30 Cold Spring Harbor Laboratory, Cold Spring Harbor, New York 11724, USA.

31 Seaver Autism Center and Dept of Psychiatry, Mount Sinai School of Medicine, New York, New York 10029, USA.

32 Department of Nanobiomedical Science, Dankook University, Cheonan 330-714, Korea.

33 Department of Biological Sciences, Dankook University, Cheonan 330-714, Korea.

34 Dept of Biological Statistics and Computational Biology, Cornell University, Ithaca, New York 14853, USA.

35 Center for Systems Biology and Dept Organismic and Evolutionary Biology, Harvard University, Cambridge, Massachusetts 02138, USA.

36 Institute of Medical Genetics, School of Medicine, Cardiff University, Heath Park, Cardiff CF14 4XN, UK.

37 Illumina, Inc., San Diego, California 92122, USA.

38 Molecular Epidemiology Section, Dept of Medical Statistics and Bioinformatics, Leiden University Medical Center 2333 ZA, The Netherlands.

39 Dept of Biological Sciences, Louisiana State University, Baton Rouge, Louisiana 70803, USA.

40 Analytic and Translational Genetics Unit, Massachusetts General Hospital, Boston, Massachusetts 02114, USA.

41 Dept of Computational Medicine and Bioinfomatics, University of Michigan, Ann Arbor, Michigan 48109, USA.

42 Dept of Anthropology, Penn State University, University Park, Pennsylvania 16802, USA.

43 Dept of Genetics, Stanford University, Stanford, California 94305, USA.

44 Ancestry.com, San Francisco, California 94107, USA.

45 Blavatnik School of Computer Science, Tel-Aviv University, 69978 Tel Aviv, Israel.

46 Dept of Microbiology, Tel-Aviv University, 69978 Tel Aviv, Israel.

47 International Computer Science Institute, Berkeley, California 94704, USA.

48 The Translational Genomics Research Institute, Phoenix, Arizona 85004, USA.

49 Dept of Human Genetics, David Geffen School of Medicine, University of California, Los Angeles, California 90024, USA.

50 Dept of Psychiatry, University of California, San Diego, La Jolla, California 92093, USA.

51 Dept of Cellular and Molecular Medicine, University of California, San Diego, La Jolla, California 92093, USA.

52 Dept of Computer Science, University of California, San Diego, La Jolla, California 92093, USA.

53 Dept of Epidemiology and Population Health, Albert Einstein College of Medicine, Bronx, New York 10461, USA.

54 Dept of Bioengineering and Therapeutic Sciences and Medicine, University of California, San Francisco, California 94158, USA.

55 Center for Biomolecular Science and Engineering, University of California, Santa Cruz, California 95064, USA.

56 Dept of Human Genetics, University of Chicago, Chicago, Illinois 60637, USA.

57 Dept of Genetics, Evolution and Environment, University College London, London WC1E 6BT, UK.

58 Dept of Genetic Medicine and Development, University of Geneva Medical School, Geneva, 1211 Switzerland.

59 Institute for Genetics and Genomics in Geneva (iGE3), University of Geneva, 1211 Geneva, Switzerland.

60 Institute for Genome Sciences, University of Maryland School of Medicine, Baltimore, Maryland 21201, USA.

61 IST/High Performance and Research Computing, University of Medicine and Dentistry of New Jersey, Newark, New Jersey 07107, USA.

62 Department of Invertebrate Zoology, American Museum of Natural History, New York 10024, USA.

63 Dept of Anthropology, University of Michigan, Ann Arbor, Michigan 48109, USA.

64 Dept of Genetics, University of North Carolina at Chapel Hill, Chapel Hill, North Carolina 27599, USA.

65 Dept of Pediatrics, University of Montréal, Ste. Justine Hospital Research Centre, Montréal, Québec H3T 1C5,Canada.

66 Dept of Biology, University of Puerto Rico, Mayagüez, Puerto Rico 00680, USA.

67 The University of Texas Health Science Center at Houston, Houston, Texas 77030, USA.

68 Eccles Institute of Human Genetics, University of Utah School of Medicine, Salt Lake City, Utah 84112, USA.

69 Dept of Genetics, Rutgers University,The State University of New Jersey, Piscataway, New Jersey 08854, USA.

70 Dept of Medicine, Division of Medical Genetics, University of Washington, Seattle, Washington 98195, USA.

71 Dept of Computer Engineering, Bilkent University, TR-06800 Bilkent, Ankara, Turkey.

72 Dept of Haematology, University of Cambridge and National Health Service Blood and Transplant, Cambridge CB2 1TN, UK.

73 Institute of Genetics and Biophysics, National Research Council (CNR), 80125 Naples, Italy.

74 Program in Computational Biology and Bioinformatics, Yale University, New Haven, Connecticut 06520, USA.

75 Dept of Computer Science, Yale University, New Haven, Connecticut 06520, USA.

76 Dept of Molecular Biophysics and Biochemistry, Yale University, New Haven, Connecticut 06520, USA.

77 Dept of Chemistry, Yale University, New Haven, Connecticut 06520, USA.

78 Dept of Chemistry and Chemical Biology, Harvard University, Cambridge, Massachusetts 02138, USA.

79 Beyster Center for Genomics of Psychiatric Diseases, University of California, San Diego, La Jolla, California 92093, USA.

80 US National Institutes of Health, National Human Genome Research Institute, 50 South Drive, Bethesda, Maryland 20892, USA.

81 Division of Allergy and Clinical Immunology, School of Medicine, Johns Hopkins University, Baltimore, Maryland 21205, USA.

82 Coriell Institute for Medical Research, Camden, New Jersey 08103, USA.

83 Centre for Health, Law and Emerging Technologies, University of Oxford, Oxford OX3 7LF, UK.

84 Genetic Alliance, London, N1 3QP, UK.

85 Johns Hopkins University School of Medicine, Baltimore, Maryland 21205, USA.

86 Dept of Medical History and Bioethics, Morgridge Institute for Research, University of Wisconsin-Madison, Madison, Wisconsin 53706, USA.

87 University of Wisconsin Law School, Madison, Wisconsin 53706, USA.

88 The Ethox Centre, Department of Public Health, University of Oxford, Old Road Campus, Oxford, OX3 7LF, UK.

89 US National Institutes of Health, Center for Research on Genomics and Global Health, National Human Genome Research Institute, 12 South Drive, Bethesda, Maryland 20892, USA.

90 Institute for Genome Sciences and Policy, Duke University, Durham, North Carolina 27708, USA.

91 Dept of Genetics, University of Pennsylvania School of Medicine, Philadelphia, Pennsylvania 19104, USA.

92 Dept of Animal Biology, Unit of Anthropology, University of Barcelona, 08028 Barcelona, Spain.

93 Cancer and Immunogenetics Laboratory, University of Oxford, John Radcliffe Hospital, Oxford OX3 9DS, UK.

94 Laboratory of Molecular Genetics, Institute of Biology, University of Antioquia, Medellín, Colombia.

95 Peking University Shenzhen Hospital, Shenzhen, 518036, China.

96 Institute of Medical Biology, Chinese Academy of Medical Sciences and Peking Union Medical College, Kunming 650118, China.

97 Instituto de Biologia Molecular y Celular del Cancer, Centro de Investigacion del Cancer/IBMCC (CSIC-USAL), Institute of Biomedical Research of Salamanca (IBSAL) & Banco Nacional de ADN Carlos III, University of Salamanca, 37007 Salamanca, Spain.

98 Instituto de Biologia Molecular y Celular del Cancer, Centro de Investigacion del Cancer/IBMCC (CSIC-USAL), Institute of Biomedical Research of Salamanca (IBSAL) & Cytometry Service and Dept of Medicine, 37007 University of Salamanca, Salamanca, Spain.

99 Ponce School of Medicine and Health Sciences, Ponce, Puerto Rico 00716, USA.

100 US National Institutes of Health, National Human Genome Research Institute, 5635 Fishers Lane, Bethesda, Maryland 20892, USA.

101 Wellcome Trust, Gibbs Building, 215 Euston Road, London NW1 2BE, UK.

102 Dept of Computer Science, Simon Fraser University, Burnaby, British Columbia V5A 1S6, Canada.

103 Dept of Bioinformatics and Computational Biology, The University of Texas MD Anderson Cancer Center, Houston, Texas 77230, USA.

104 Swiss Institute of Bioinformatics, 1211 Geneva, Switzerland.

105 Dipartimento di Scienze Biomediche, Università delgi Studi di Sassari, 07100 Sassari, Italy.

106 Istituto di Ricerca Genetica e Biomedica, CNR, Monserrato, 09042 Cagliari, Italy.

107 Center for Advanced Studies, Research, and Development in Sardinia (CRS4), AGCT Program, Parco Scientifico e tecnologico della Sardegna, 09010 Pula, Italy.

108 University of Michigan Sequencing Core, University of Michigan, Ann Arbor, Michigan 48109, USA.

109 National Institute on Aging, Laboratory of Genetics, Baltimore, Maryland 21224,USA.

110 Life Technologies, Beverly, Massachusetts 01915, USA.

111 Howard Hughes Medical Institute, Santa Cruz, California 95064, USA.

‡ Deceased
